# Supplementary material for: Synthetic NAC 71-82 Peptides Designed to Produce Fibrils with Different Protofilament Interface Contacts
Source: Int J Mol Sci. 2021 Aug 28;22(17):9334. doi: 10.3390/ijms22179334 (PMC8431055; doi:10.3390/ijms22179334)
Supplement: Supplementary file 1 [file ijms-22-09334-s001.zip › ijms-1354644-supplementary.pdf]

Supporting information for:

Synthetic NAC 71-82 peptides designed to produce fibrils with different protofilament interface contacts

Thomas Näsström,[a] Tobias Dahlberg,[b] Dmitry Malyshev,[b] Jörgen Ådén,[c] Per Ola Andersson,[d] Magnus Andersson,[b] Björn C. G. Karlsson†[a]

[a] *Physical Pharmacy Laboratory, Linnaeus University Centre for Biomaterials Chemistry, Linnaeus University, SE-392 31 Kalmar, Sweden.* [b] *The Biophysics and Biophotonics Group. Department of Physics, Umeå University, SE-901 87 Umeå, Sweden.* [c] *Department of Chemistry, Umeå University, SE-901 87 Umeå, Sweden.* [d] *Department of Material Science and Engineering: Applied Materials Science, Uppsala University, SE-751 03, Uppsala, Sweden.*

† *Correspondence to: bjorn.karlsson@lnu.se*

TABLE OF CONTENTS	NAME	PAGE(S)
**Molecular dynamics (MD) simulation**		
Peptide aggregation protocol		2-4
Peptide aggregation snapshots	Figure S1	5
Peptide aggregation kinetics:		
- *P2-L(G,G)-CT*	Figure S2A	6
- *P2-L(G,G)*	Figure S2B	6
Solvent accessible surface area (SASA) over time	Figure S3	7
Peptide aggregate structures:		
- *P2-L(G,G)-CT*	Figure S4A	8
- *P2-L(G,G)*	Figure S4B	8
Peptide aggregate shape descriptors:		
- *P2-L(G,G)-CT*	Table S1A	9
- *P2-L(G,G)*	Table S1B	10
Radius of gyration (R_g) over time	Figure S5	11
Tail-to-tail distance histograms for each simulation system	Figure S6	12
Tail-to-tail distance histograms for all simulation systems	Figure S7	12
**Fibril formation kinetics**	**Figure S8**	**13**
**Circular dichroism (CD) spectroscopic analysis of the soluble fractions of fibrillar mixtures**	**Figure S9**	**14**
**Example Raman spectra**	**Figure S10**	**15**
**TEM analysis of fibrils generated after 72 h of incubation:**		
- *P2-L(G,G)–water*	Figure S11	16
- *P2-L(G,G)-CT–20 mM Tris-HCl and 0.15 M NaCl*	Figure S12	17
- *P1–water*	Figure S13	18
- *P1-CT–20 mM Tris-HCl and 0.15 M NaCl*	Figure S14	19
- *Quantification of fibrillar widths (nm)*	Table S2	20

Molecular dynamics (MD) simulation

Peptide aggregation protocol

Generation of pre-folded single peptide starting structures

Single extended structures of the double NAC 71-82 fragment P2-L(G,G) and P2-L(G,G)-CT peptides were built using the XLEaP module and the Amber14SB force field [1] implemented in the AmberTools19 software (v. 19, USCF, San Francisco, CA, USA) [2]. A series of energy minimisation and molecular dynamics (MD) steps were then performed to generate pre-folded structures to be used in peptide aggregation simulations. Energy minimisation was performed using 500 steps of the steepest descent and 500 steps of the conjugate gradient algorithm. Totally 20 ps of MD simulation were then performed on each peptide system to slowly increase the temperature from 0 to 200 K using the Berendsen thermostat and a 1.0 ps temperature coupling constant. Both energy minimisation and subsequent MD simulation were performed using non-periodic conditions and the standard pairwise Born implicit solvent model (igb=1). A 999 Å cut-off for non-bonded interactions was used and the time-step during MD was set to 0.5fs, constraining all bonds to hydrogen using the SHAKE algorithm.

Building multiple peptide aggregation systems

The folded structure obtained after 20 ps of MD simulation was extracted and used as a starting peptide conformation in multiple peptide aggregation simulations. Initially 20 copies of each peptide and 40 neutralising chloride ions were randomly mixed using a 10 Å distance tolerance in a cube in which one side was 170 Å in PACKMOL (v. 18.169, Universities of Campinas and São Paulo, Brazil) [3]. Additional 290 chloride and 290 sodium ions were added to the P2-L(G,G)-CT system to reach a physiological salt concentration (0.15 M). In a second packing step, 100,000 water molecules were added to each peptide system. To improve statistical sampling, totally five replicate systems were generated for each type of peptide using random seed numbers (seed -1) in PACKMOL during molecular packing.

Multiple peptide aggregation simulations

MD simulations on systems prepared by PACKMOL were performed using a previously described protocol for the analysis of aggregation of the single NAC 71-82 peptides [4]. After a series of initial energy minimisation and equilibration stages, 200 ns of trajectory data from simulation at NPT condition (1 bar and 310.15 K) was collected for each system resulting in totally 1 ms being collected for the P2-L(G,G) and the P2-L(G,G)-CT peptide

system, respectively. All MD simulations were performed using the AMBER17 software (v.16, UCSF, San Francisco, CA) [5]. The TIP3P water model was used for explicit water and the ion parameters used were those derived from Joung and Cheatham [6].

Peptide aggregation analyses

Peptide aggregate size distributions were calculated using the *gmx clustsize* program of GROMACS (v. 2019.2) [7]. A peptide aggregate was considered to be present when atomic contacts between any atom of interacting peptides were less than 3.5 Å. The aggregation propensity, *AP*, in each simulation was calculated according to Equation 1 where $SASA_{max}$ is the solvent accessible surface area (SASA) for 20 fully extended peptides (61600 $Å^2$ or 58880 $Å^2$ for P2-L(G,G) and P2-L(G,G)-CT, respectively) whereas $SASA_i$ is the SASA of all peptides at frame, *i*. All SASA values were calculated using the linear combination of pairwise overlaps (LCPO) surface area method [8] using a van der Waals offset of 1.4 Å.

$$AP=\frac{SASA_{max}}{SASA_i} \qquad \text{Equation 1}$$

The compactness of a peptide aggregate was investigated by calculating the factor *F*, Equation 2, which is a ratio between the radius of gyration, R_g, and the radius of gyration for a peptide aggregate with radius based on a perfect spherical shape, R_g', Equation 3.

$$F=\frac{R_g}{R_g'} \qquad \text{Equation 2}$$

$$R_g'=\sqrt[3]{\frac{3\,N_{pep}V_{pep}}{4\pi}} \qquad \text{Equation 3}$$

In this equation, N_{pep} is the number of peptides in an aggregate and V_{pep} is the total volume of a single peptide (2132 $Å^3$ and 2032 $Å^3$ for P2-L(G,G) and P2-L(G,G)-CT, respectively). The relative shape anisotropy, κ^2, was calculated using the gyration tensor, λ, of the *x*,*y* and *z* dimensions and the following criteria, $\lambda_x > \lambda_y > \lambda_z$. The radius of gyration, R_g, was calculated from Equation 4 using the following relation:

$$R_g^2 = \lambda_x + \lambda_y + \lambda_z \qquad \text{Equation 4}$$

The shape of the observed peptide aggregates was extracted from a range of shape descriptors. Relative asphericity, *b*, and the acylindricity, *c*, values, which indicate the degree of deviation of a spherical or cylindrical shape of a peptide aggregate, were calculated from Equations 5 and 6, respectively.

$$b = \frac{\lambda_x - 0.5\,(\lambda_y + \lambda_z)}{R_g^2} \qquad \text{Equation 5}$$

$$c = \frac{\lambda_y - \lambda_z}{R_g^2} \qquad \text{Equation 6}$$

The relative shape anisotropy, κ^2, can adopt values between zero and one where the value of zero can be associated with the formation of *e.g.*, a perfectly spherical aggregate and the value of one is attributed to a perfectly linear chain, Equation 7:

$$\kappa^2 = 1 - 3\,\frac{(\lambda_x \lambda_y + \lambda_x \lambda_z + \lambda_y \lambda_z)}{(\lambda_x + \lambda_y + \lambda_z)^2} \qquad \text{Equation 7}$$

Finally, peptide tail-to-tail (T-T) distances were calculated using the atomic distance between the backbone carbonyl carbon of the first and last valine amino acid residue of each peptide. The relative backbone length of each peptide, $R_{T\text{-}T}$, was calculated as a ratio of the measured TT of the carbonyl carbon atoms of the first and last valine amino acid residue of a peptide and the backbone length of a fully extended structure, Equation 8. R_0 for both P2-L(G,G) and P2-L(G,G)-CT peptides were measured to be 91.03 Å.

$$R_{T\text{-}T} = \frac{R}{R_0} \qquad \text{Equation 8}$$

All calculated data from MD simulation trajectories are presented as mean ± standard deviation, if not otherwise stated. Trajectories were visualised and snapshots were extracted using the VMD software (v. 1.9.2, University of Illinois at Urbana-Champaign, IL, USA) [9].

References

1. Maier, J.A.; Martinez, C.; Kasavajhala, K.; Wickstrom, L.; Hauser, K.E.; Simmerling, C. Ff14SB: Improving the Accuracy of Protein Side Chain and Backbone Parameters from Ff99SB. *J. Chem. Theory Comput.* **2015**, *11*, 3696–3713, doi:10.1021/acs.jctc.5b00255.

2. Roe, D.R.; Cheatham, T.E. PTRAJ and CPPTRAJ: Software for Processing and Analysis of Molecular Dynamics Trajectory Data. *J. Chem. Theory Comput.* **2013**, *9*, 3084–3095, doi:10.1021/ct400341p.
3. Martínez, L.; Andrade, R.; Birgin, E.G.; Martínez, J.M. PACKMOL: A Package for Building Initial Configurations for Molecular Dynamics Simulations. *J. Comput. Chem.* **2009**, *30*, 2157–2164, doi:10.1002/jcc.21224.
4. Näsström, T.; Andersson, P.O.; Lejon, C.; Karlsson, B.C.G. Amyloid Fibrils Prepared Using an Acetylated and Methyl Amidated Peptide Model of the α-Synuclein NAC 71-82 Amino Acid Stretch Contain an Additional Cross-β Structure Also Found in Prion Proteins. *Sci. Rep.* **2019**, *9*, 15949, doi:10.1038/s41598-019-52206-5.
5. Case, D.A.; Betz, R.M.; Cerutti, D.S.; Cheatham, T.E. (III); Darden, T.A.; Duke, R.E.; Giese, T.J.; Gohlke, H.; Goetz, A.W.; Homeyer, N.; et al. *AMBER17*; University of California, San Francisco, USA, 2017;
6. Joung, I.S.; Cheatham, T.E. Determination of Alkali and Halide Monovalent Ion Parameters for Use in Explicitly Solvated Biomolecular Simulations. *J. Phys. Chem. B* **2008**, *112*, 9020–9041, doi:10.1021/jp8001614.
7. Abraham, M.J.; van der Spoel, D.; Lindahl, E.; Hess, B.; and the GROMACS development team *GROMACS User Manual Version 2019.2*;
8. Weiser, J.; Shenkin, P.; Still, W.C. Approximate Atomic Surfaces from Linear Combinations of Pairwise Overlaps (LCPO). *J. Comput. Chem.* **1999**, doi:10.1002/(SICI)1096-987X(19990130)20:2%3C217::AID-JCC4%3E3.0.CO;2-A.
9. Humphrey, W.; Dalke, A.; Schulten, K. VMD: Visual Molecular Dynamics. *J. Mol. Graph.* **1996**, *14*, 33–8, 27–8.

Peptide aggregation snapshots

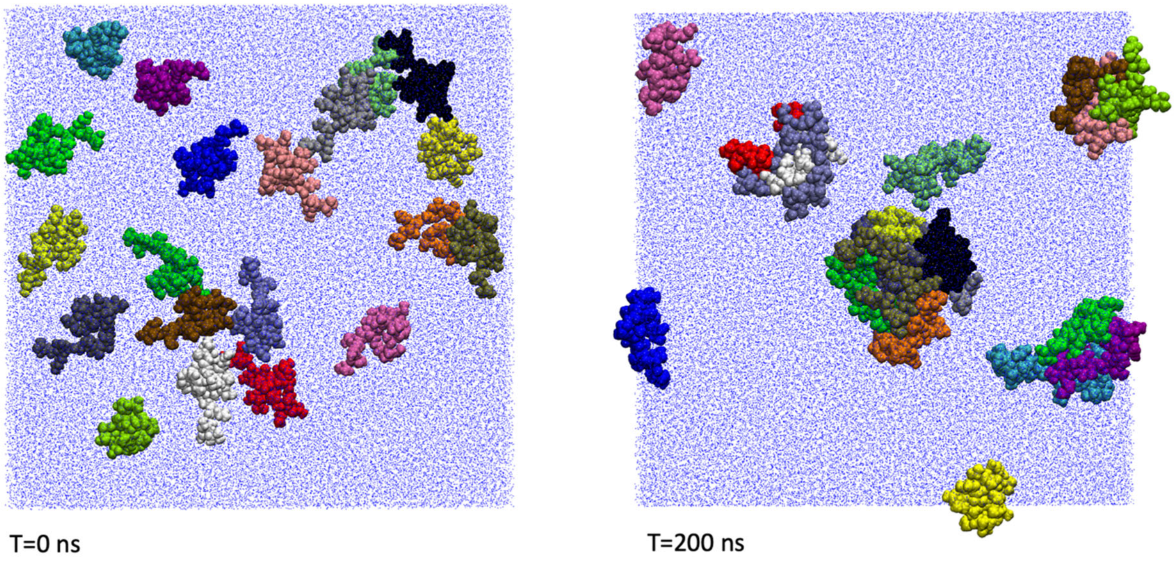

Figure S1 A selected example (P2-L(G,G)) of the peptide aggregation simulation protocol used in this work. Prior to simulation (t=0 ns), 20 peptides were initially randomly distributed using a 10 Å minimum atomic peptide-peptide distance in a 170 x 170 x 170 Å cubic box. Peptides were thereafter mixed with 100,000 water molecules. A total number of 200 ns of trajectory data was collected in each simulation system. Here, each peptide in the simulation box is depicted using different colours and van der Waals representation. Water oxygen is shown as blue dots.

Peptide aggregation kinetics

P2-L(G,G)-CT

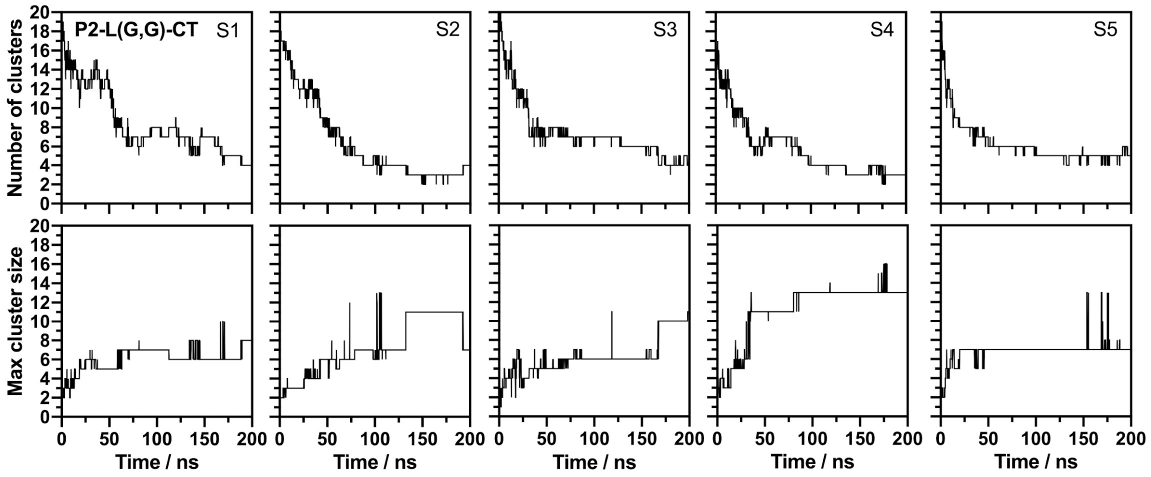

Figure S2A Number of clusters (top panels) and the maximum cluster size (bottom panels) formed over time in each simulation replica (S1-S5) of the P2-L(G,G)-CT system.

P2-L(G,G)

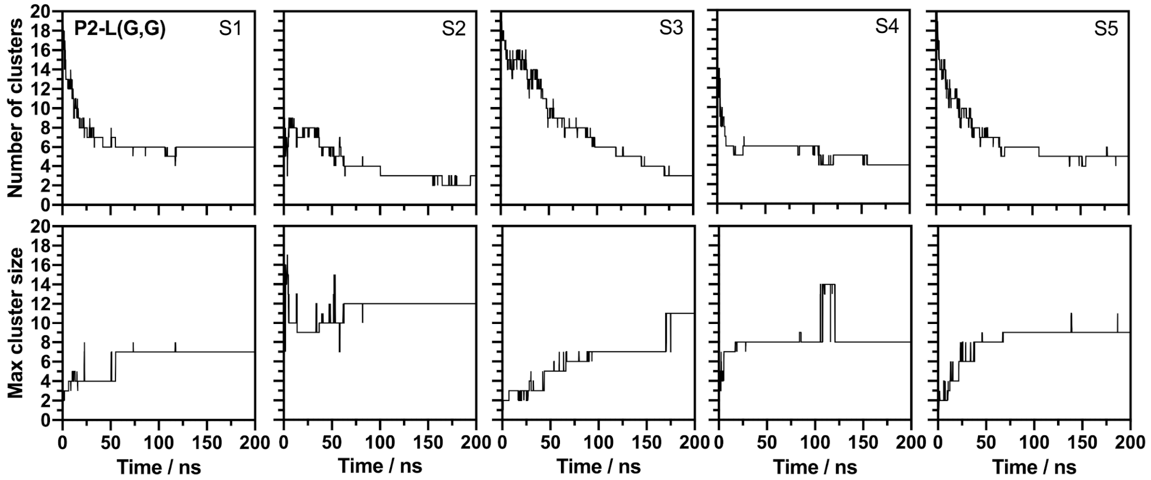

Figure S2B Number of clusters (top panels) and the maximum cluster size (bottom panels) formed over time in each simulation replica (S1-S5) of the P2-L(G,G) system.

Solvent accessible surface area (SASA) over time

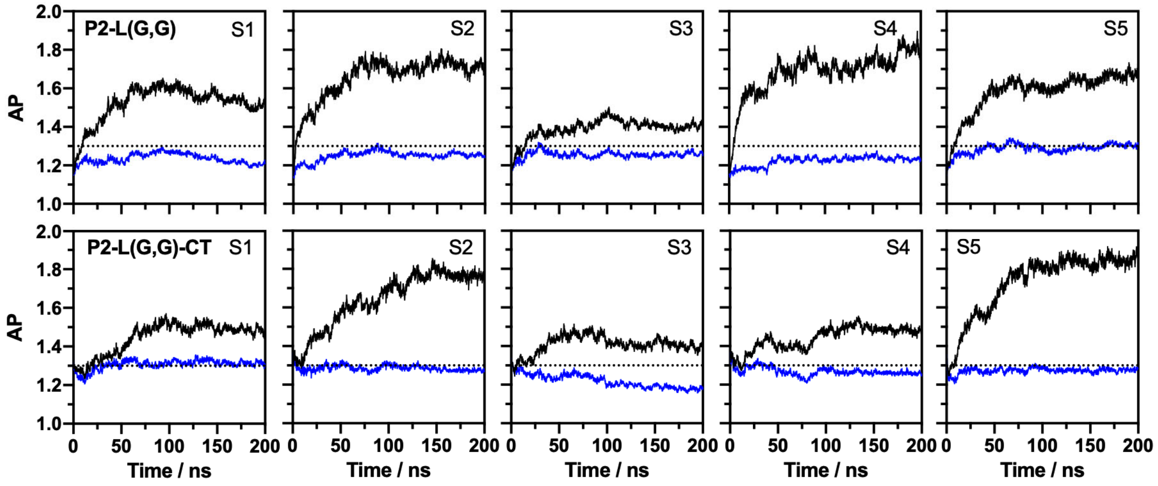

Figure S3 Aggregation propensities, *AP*, over time in each simulation replica (S1-S5) of the P2-L(G,G) (top panels) or the P2-L(G,G)-CT (bottom panels) (bottom panels). Black lines describe the total decrease of SASA (aggregation + folding) whereas the blue line describe the change in SASA due to peptide folding only. The inserted dashed line denotes the maximum change in SASA due to folding (*AP* = 1.3).

Peptide aggregate structures

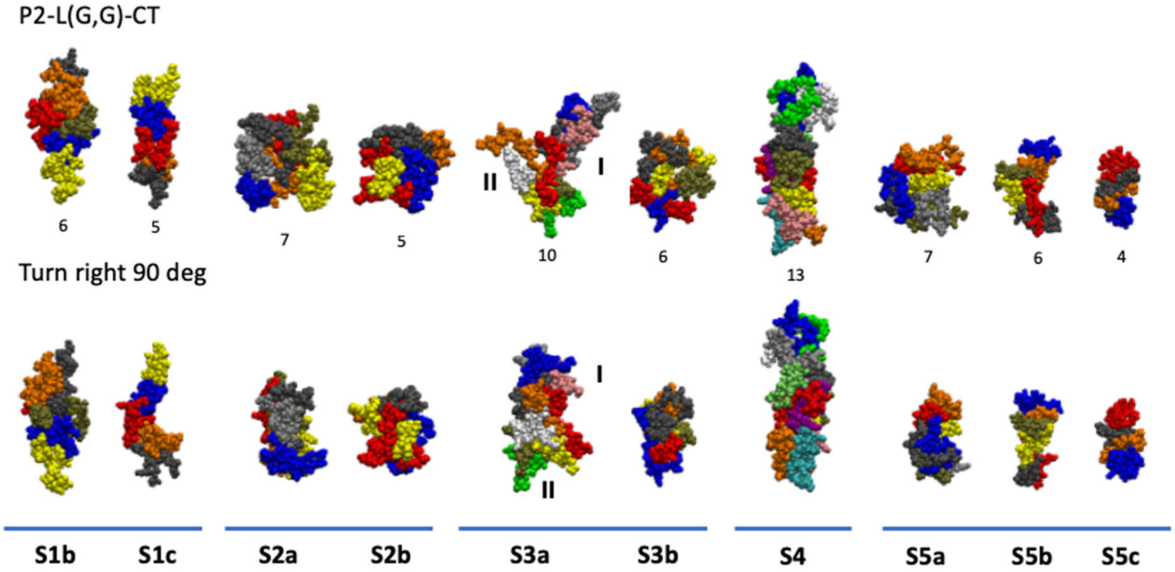

Figure S4A Peptide aggregate structures observed in the P2-L(G,G)-CT simulation replicas (S1-S5). Here, each peptide in each aggregate is depicted using different colours and van der Waals representation.

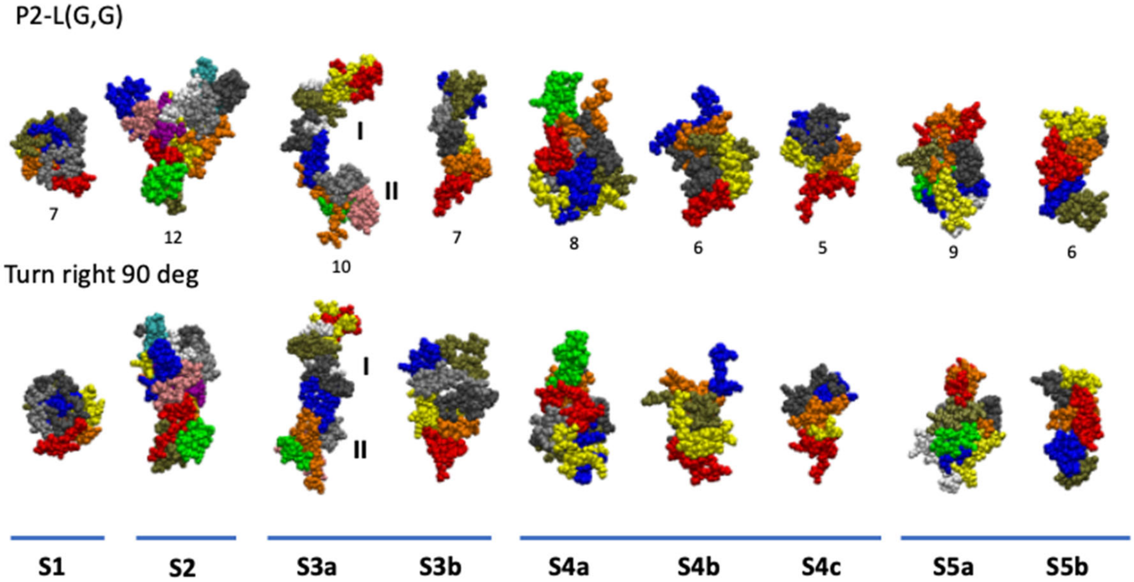

Figure S4B Peptide aggregate structures observed in the P2-L(G,G) simulation replicas (S1-S5). Here, each peptide in each aggregate is depicted using different colours and van der Waals representation.

Peptide aggregate shape descriptors

P2-L(G,G)-CT

Table S1A Analyses of the size (number of peptides, N_{pep}) and shape of the largest stable peptide aggregates formed at different time-points (Agg./ns) during P2-L(G,G)-CT MD aggregation simulations (Sim). All values (when possible) are presented as mean ± standard deviation. Peptide aggregates demonstrating nano fibre properties have been marked out in yellow. See information on pages 3 – 4 for detailed explanation on the factors presented below.

Sim	$N_{pep.}$	Agg. /ns	R_g/Å[a]	F	κ^2	b	c	L_X/L_Z	L_Y/L_Z
S1	a-8	>195	-	-	-	-	-	-	-
	b-6	80-	16.8± 0.6	1.17±0.04	0.14±0.02	0.34±0.03	0.17±0.02	4.1±0.6	2.2±0.3
	c-5	170-	18.9± 0.5	1.40±0.04	0.14±0.02	0.30±0.03	0.24±0.02	4.9±0.7	3.3±0.4
S2	a-7	130-	18.1± 0.2	1.19±0.01	0.10±0.00	0.19±0.03	0.28±0.02	3.5±0.3	3.2±0.3
	b-5	90-	15.1± 0.2	1.11±0.02	0.05±0.01	0.20±0.03	0.11±0.03	2.2±0.3	1.5± 0.1
S3	a-10(11)	170-	24.7± 0.9	1.44±0.05	0.08±0.01	0.20±0.06	0.22±0.05	3.1±0.4	2.5±0.4
	I-6	75-	18.0± 0.5	1.25±0.04	0.02±0.01	0.12±0.04	0.07±0.06	1.6±0.2	1.3±0.2
	II-4	30-	14.6± 0.4	1.16±0.03	0.14±0.04	0.36±0.05	0.10±0.03	3.2±0.8	2.3±0.4
	b-6	130-	18.1± 0.9	1.26±0.06	0.09±0.04	0.23±0.10	0.23±0.05	3.2±0.8	2.3±0.4
S4	13	90-	26.0± 0.3	1.39±0.01	0.29±0.01	0.54±0.00	0.03±0.00	5.0±0.3	1.2±0.1
S5	a-7	55-	17.5± 0.2	1.15±0.01	0.06±0.00	0.16±0.03	0.20±0.02	2.4±0.1	2.1±0.1
	b-6	55-	19.5± 0.4	1.35±0.03	0.16±0.03	0.39±0.04	0.00±0.03	3.0±0.5	1.0±0.2
	c-4	100-	13.8± 0.3	1.10±0.03	0.09±0.03	0.29±0.06	0.06±0.04	2.6±0.5	1.3±0.2

[a] During the calculation of the radius of gyration, R_g, the value of the volume of the peptide was $V_{P2-L(G,G)-CT}$ = 2091 Å^3.

P2-L(G,G)

Table S1B Analyses of the size (number of peptides, N_{pep}) and shape of the largest stable peptide aggregates formed at different time-points (Agg./ns) during P2-L(G,G) MD aggregation simulations (Sim). All values (when possible) are presented as mean ± standard deviation. Peptide aggregates demonstrating nano fibre properties have been marked out in yellow. See information on pages 3 – 4 for detailed explanation on the factors presented below.

Sim	N_{pep}	Agg./ ns	R_g/Å[a]	F	κ^2	b	c	L_X/L_Z	L_Y/L_Z
S1	7	60-	16.1± 0.2	1.06±0.01	0.01±0.00	0.09±0.02	0.02±0.03	1.3±0.1	1.1±0.1
S2	12	140-	24.1± 0.3	1.31±0.02	0.06±0.01	0.43±0.02	0.21±0.03	2.4±0.3	2.2±0.3
S3	a-10(11)*	185-	31.7± 1.0	1.84±0.06	0.02±0.01	0.09±0.03	0.13±0.05	1.7±0.3	1.6±0.3
	I-(6)	100-	22.0± 0.8	1.51±0.06	0.13±0.03	0.33±0.05	0.17±0.03	4.0±0.7	2.2±0.3
	II-(4)	160-	15.1± 0.3	1.19±0.03	0.11±0.02	0.32±0.04	0.04±0.06	2.7±0.5	1.3±0.3
	b-7	90-	19.3± 0.7	1.27±0.04	0.23±0.06	0.47±0.07	0.11±0.02	5.5±1.2	2.0±0.2
S4	a-8	50-	18.6± 0.2	1.17±0.01	0.03±0.00	0.07±0.01	0.20±0.02	2.0±0.1	1.8±0.1
	b-6	130-	16.2± 0.4	1.12±0.03	0.06±0.01	0.13±0.03	0.23±0.03	2.4±0.3	2.3±0.3
	c-5	165-	16.7± 0.9	1.22±0.06	0.19±0.06	0.43±0.08	0.03±0.03	3.7±0.8	1.2±0.2
S5	a-9	100-	19.6± 0.4	1.18±0.02	0.07±0.01	0.27±0.03	0.03±0.04	2.3±0.2	1.2±0.2
	b-6*	130-	18.0± 0.3	1.24±0.02	0.13±0.01	0.36±0.02	0.04±0.02	1.9±0.2	1.1±0.1

[a] During the calculation of the radius of gyration, R_g, the value of the volume of the peptide was $V_{P2\text{-}L(G,G)} = 2132$ Å^3 * Values were calculated only for the last 15 ns of the 200 ns simulation time.

Radius of gyration (R_g) over time

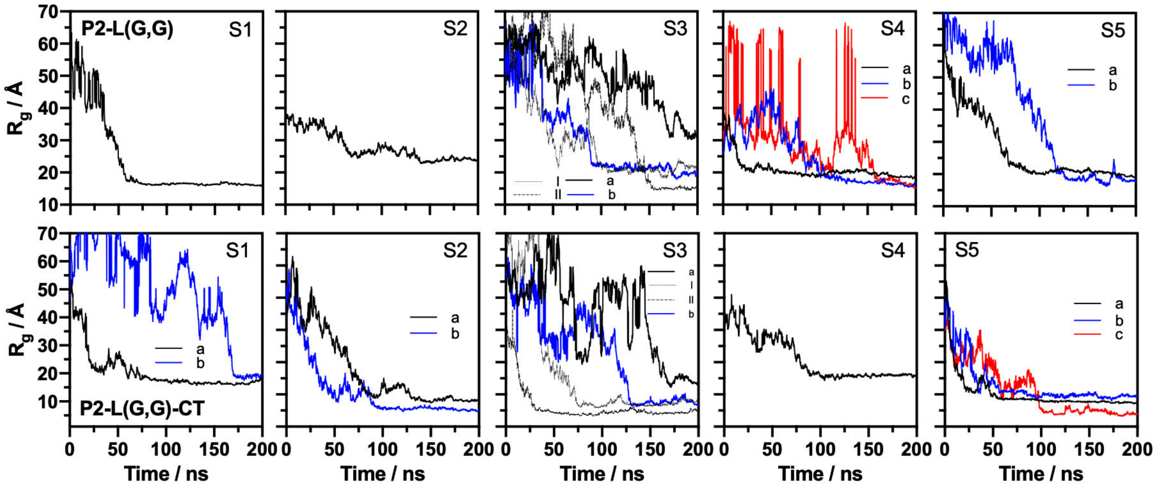

Figure S5 Radius of gyration, R_g, over time for studied peptide aggregates found in each simulation replica (S1-S5). Inserted letters in each panel denote the aggregate analysed. The structure and shape descriptors for each of these aggregates are presented in Figures S4AB and Tables S1AB.

Tail-to-tail distance histograms for each simulation system

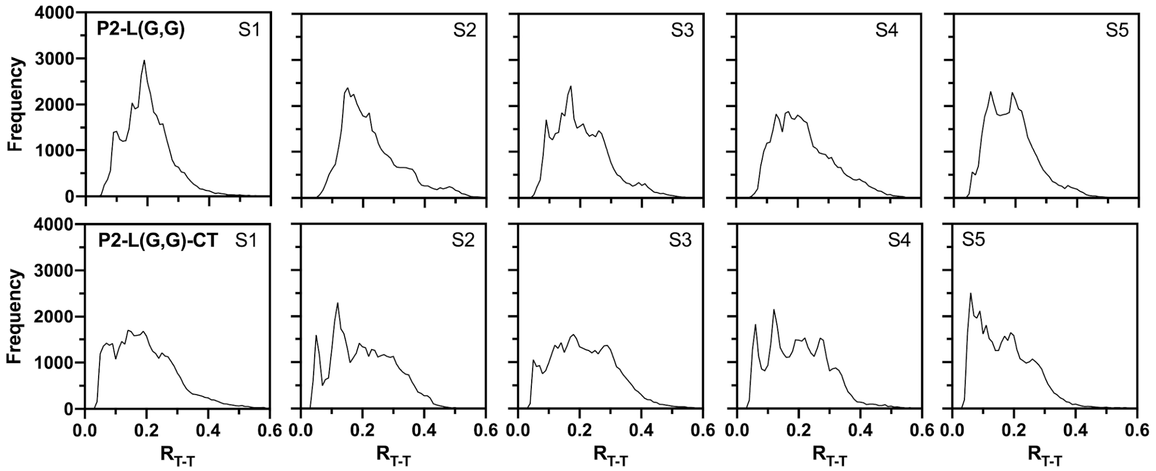

Figure S6 Relative tail-to-tail distance, R_{T-T}, histograms calculated for each separate simulation replica (S1-S5) of the P2-L(G,G) (top panels) or the P2-L(G,G)-CT (bottom panels) systems. R_0=91.03 Å.

Tail-to-tail distance histograms for all simulation systems

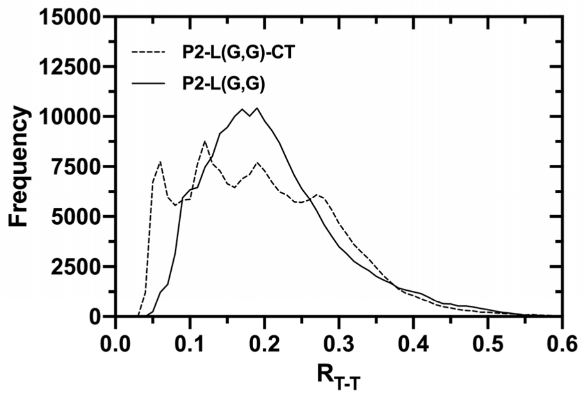

Figure S7 The total histogram of all relative tail-to-tail distances, R_{T-T}, found in each simulation replica (S1-S5) of the P2-L(G,G) (solid line) or the P2-L(G,G)-CT (dashed line).

Fibril formation kinetics

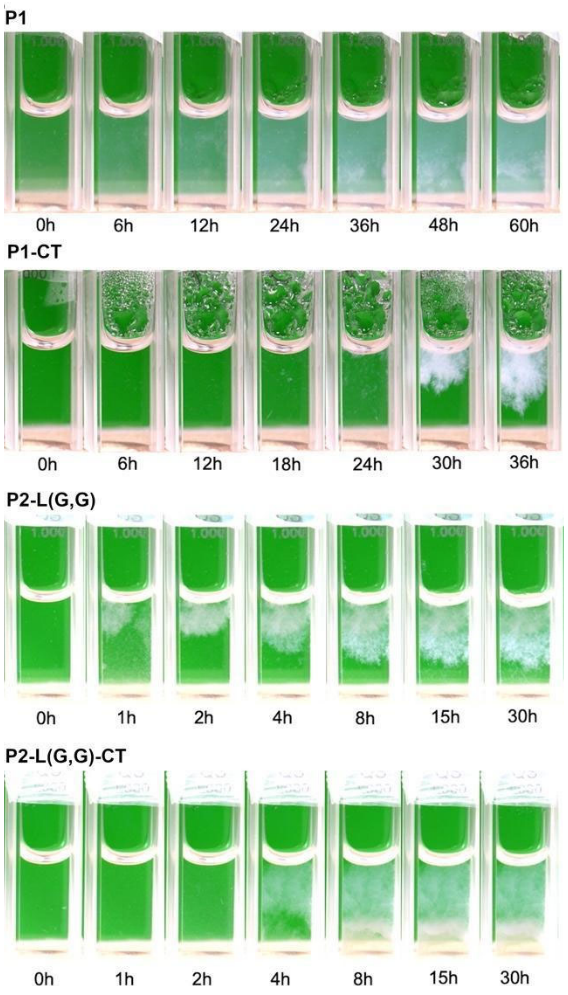

Figure S8 Fibrillisation of peptides reveal different aggregation kinetics and structural diversity. Panels shown represent snapshots captured of fibrillisation mixtures of single (P1 and P1-CT) or double (P1-L(G,G) and P1-L(G,G)-CT) fragments of the NAC 71-82 aa stretch of ⊚-synuclein captured at different time points. For a detailed description of the amino acid sequence of each peptide see **Table 2** of the main paper.

Circular dichroism (CD) spectroscopic analysis of the soluble fractions of fibrillar mixtures

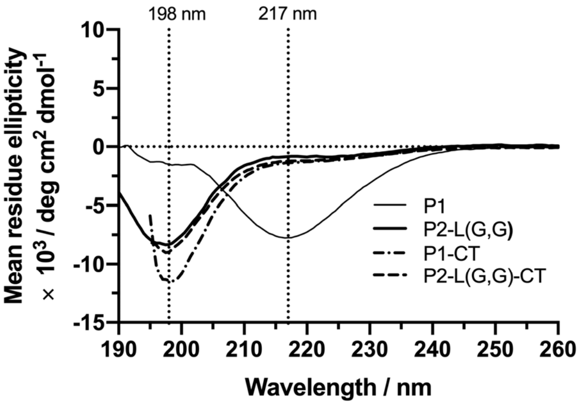

Figure S9 CD spectra of peptides found in the fibrillisation mixture supernatants. Curves presented show the calculated mean residue ellipticities between 190-260 nm for the single P1-CT or between 195-260 nm for double (P2-L(G,G) and P2-L(G,G)-CT) peptide fragments of the NAC 71-82 aa stretch of ⊚-synuclein. The CD spectrum of the single peptide fragment P1 from a fibrillisation supernatant has been reported previously (Näsström, T. *et al. Int. J. Mol. Sci.* **2020**, *21*, 1629) and inserted in this figure as a comparison. For a detailed description of the amino acid sequence of each peptide see **Table 2** of the main paper.

Example Raman spectra

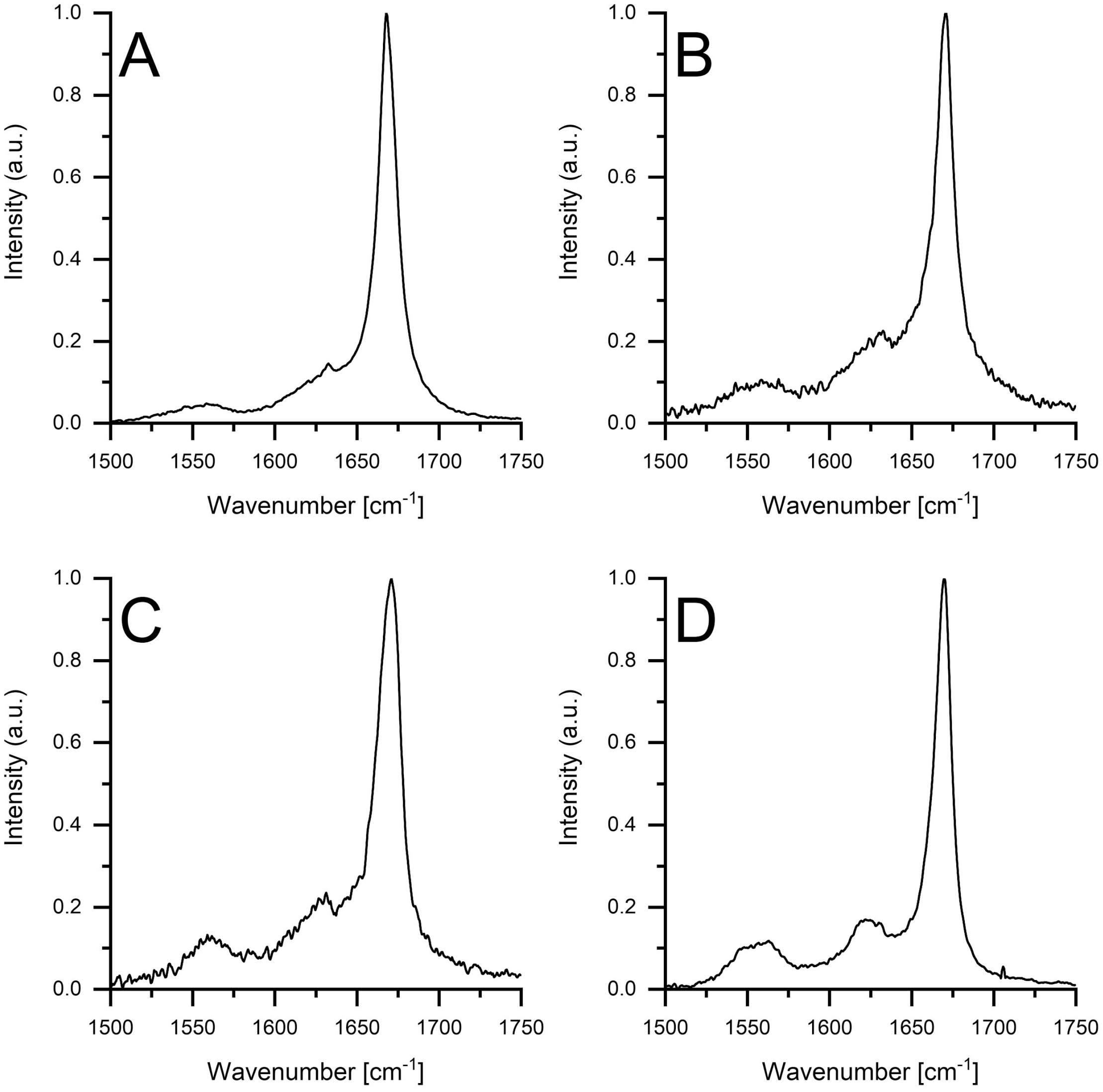

Figure S10 Averaged Raman spectra (n=5) from samples in P1 **A,** P1-CT **B**, P2-L(G,G) **C**, and P2-L(G,G)-CT **D**.

TEM analysis of fibrils generated after 72 h of incubation

P2-L(G,G) – water

A B

C D

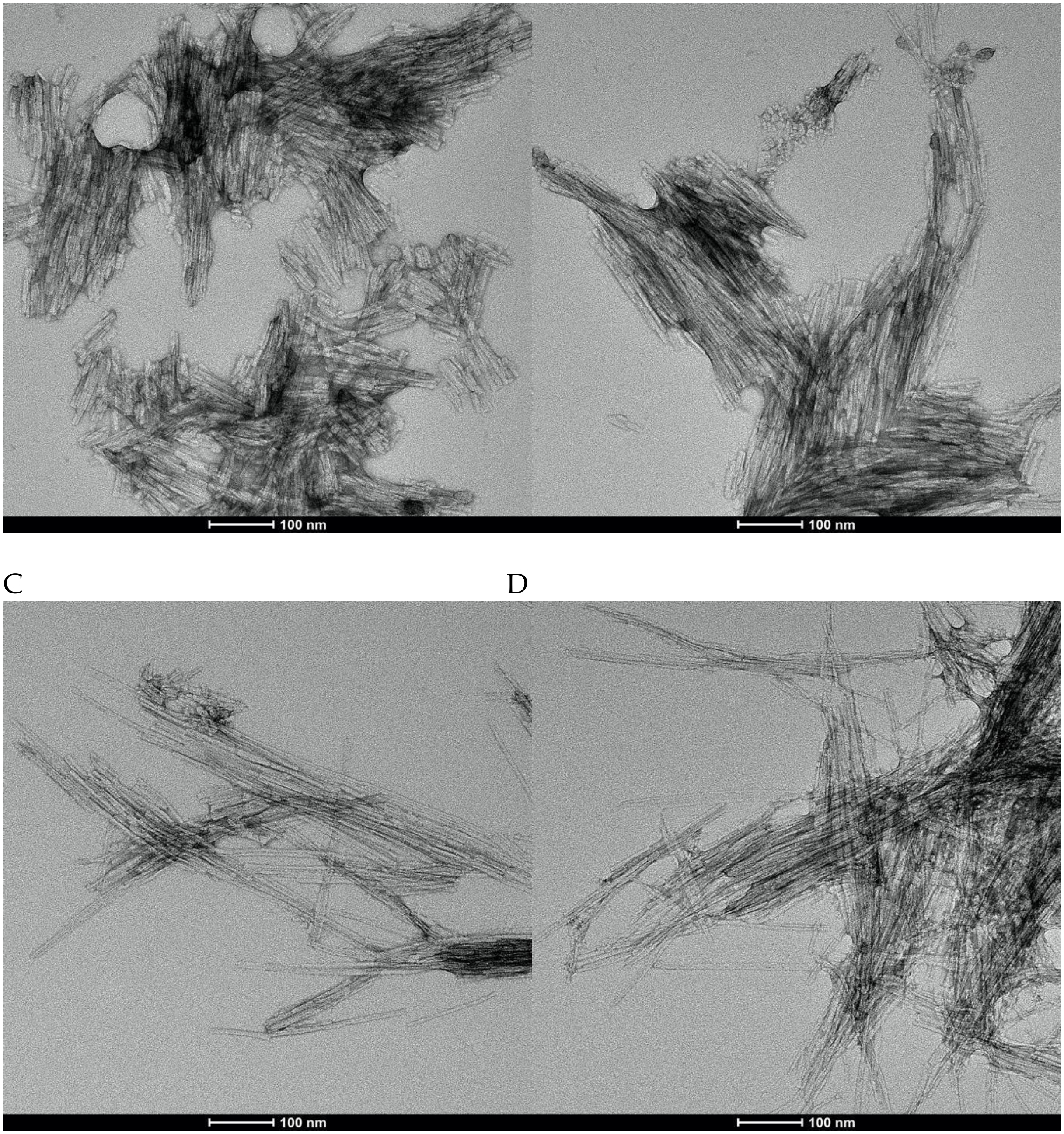

Figure S11 P2-L(G,G) fibrils (A-D) as visualised by TEM. Scale bars represent 100 nm.

P2-L(G,G)-CT – 20 mM Tris-HCl buffer and 0.15 M NaCl

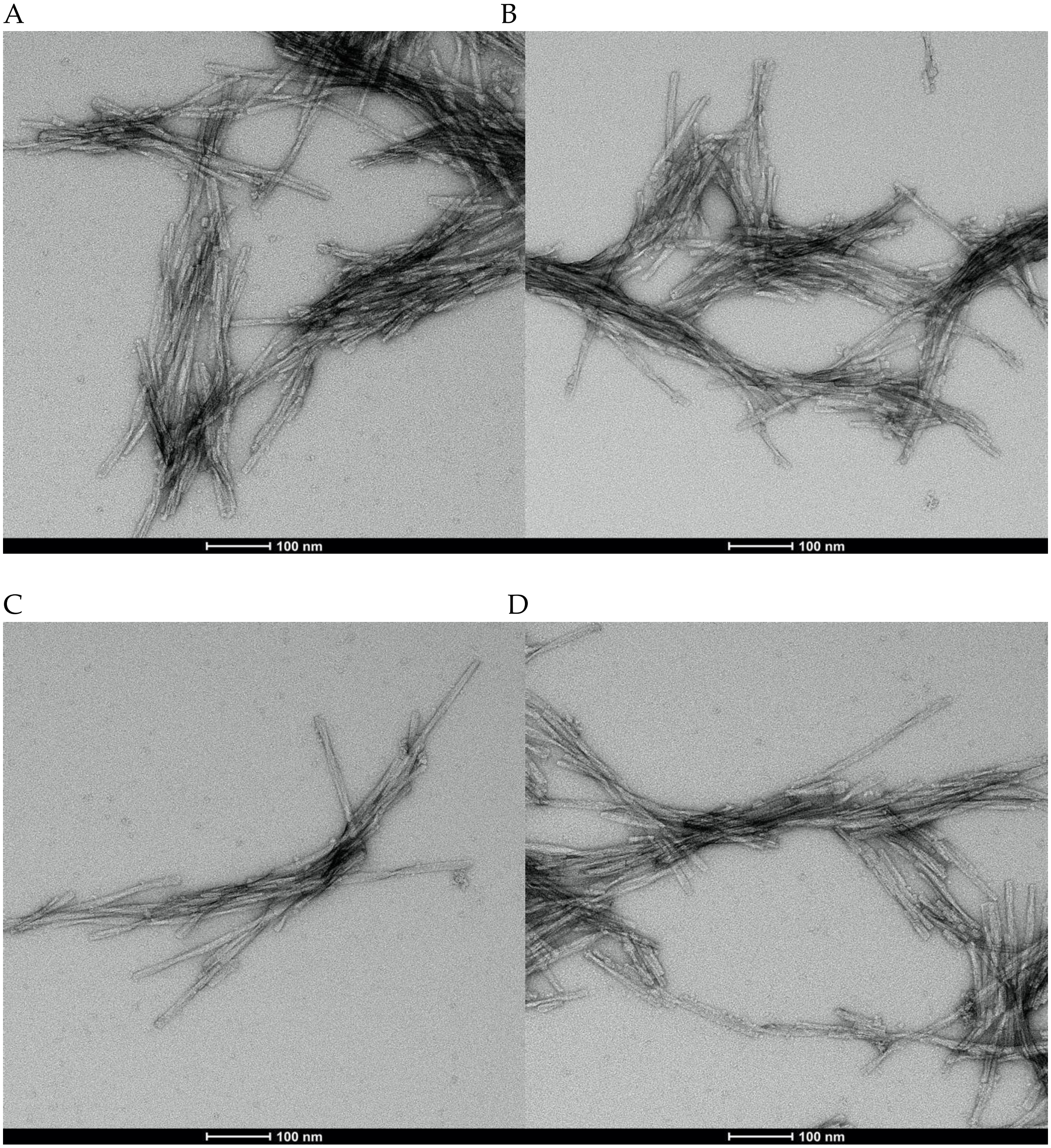

Figure S12 P2-L(G,G)-CT fibrils (A-D) as visualised by TEM. Scale bars represent 100 nm.

P1 - water

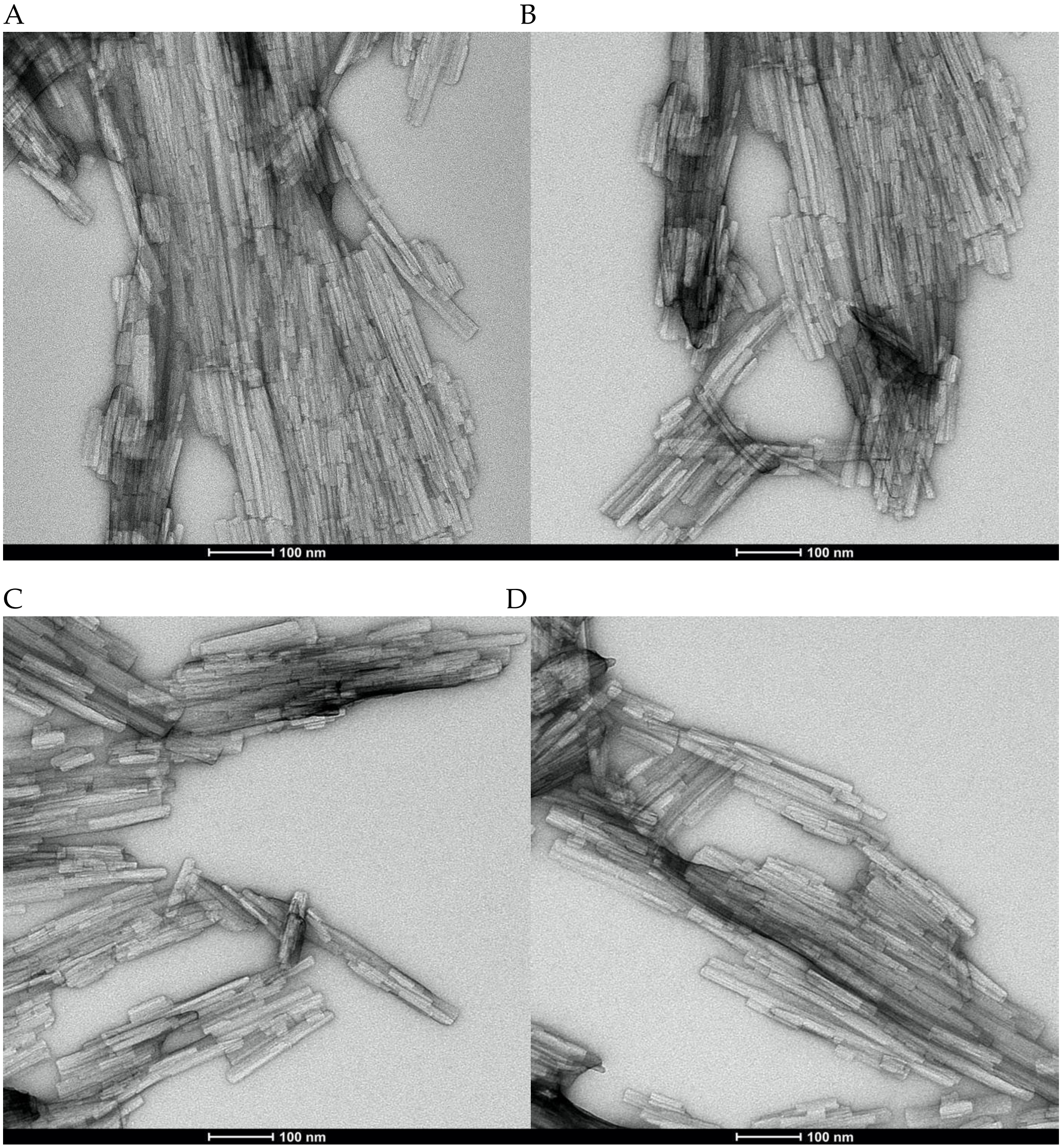

Figure S13 P1 fibrils (A-D) as visualised by TEM. Scale bars represent 100 nm.

P1-CT – 20 mM Tris-HCl buffer and 0.15 M NaCl

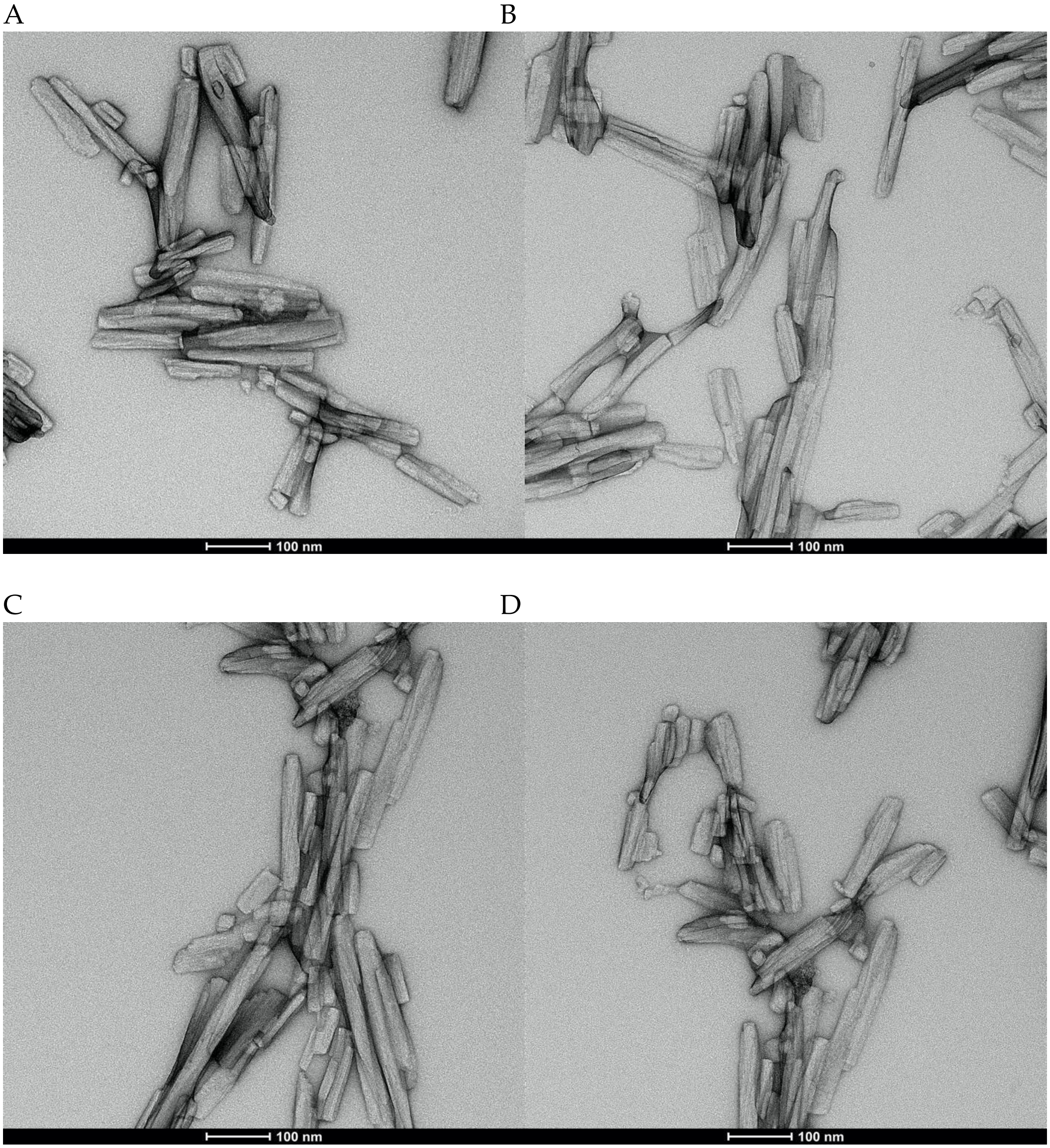

Figure S14 P1-CT fibrils (A-D) as visualised by TEM. Scale bars represent 100 nm.

Quantification of fibrillar widths (nm)

Table S2 Measurement of fibrillar widths (nm) using the Image J software (v. 1.52q)

	Widths (nm)	
Objects measured	P2-L(G,G) CT	P2-L(G,G)-
1	5.0	10.4
2	5.1	10.5
3	5.1	10.4
4	10.3	10.0
5	10.1	10.3
6	10.2	10.2
